# Review and Evaluation of MAC Protocols for Satellite IoT Systems Using Nanosatellites

**DOI:** 10.3390/s19081947

**Published:** 2019-04-25

**Authors:** Tomás Ferrer, Sandra Céspedes, Alex Becerra

**Affiliations:** 1 Department of Electrical Engineering, Universidad de Chile, Av. Tupper 2007, Santiago 8370451, Chile; tomas.ferrer@ing.uchile.cl; 2 NIC Chile Research Labs, Universidad de Chile, Santiago 8370403, Chile; 3 Aurora Space, Santiago 7750053, Chile; abecerra@auroraspace.cl

**Keywords:** CubeSats, internet of things, medium access control, nanosatellites, sensor networks, wireless access networks

## Abstract

Extending the internet of things (IoT) networks to remote areas under extreme conditions or for serving sometimes unpredictable mobile applications has increased the need for satellite technology to provide effective connectivity. However, existent medium access control (MAC) protocols deployed in commercial satellite networks were not designed to offer scalable solutions for the increasing number of devices predicted for IoT in the near future, nor do they consider other specific IoT characteristics. In particular, CubeSats—a low-cost solution for space technology—have the potential to become a wireless access network for the IoT, if additional requirements, including simplicity and low demands in processing, storage, and energy consumption are incorporated into MAC protocol design for satellite IoT systems. Here we review MAC protocols employed or proposed for satellite systems and evaluate their performance considering the IoT scenario along with the trend of using CubeSats for IoT connectivity. Criteria include channel load, throughput, energy efficiency, and complexity. We have found that Aloha-based protocols and interference cancellation-based protocols stand out on some of the performance metrics. However, the tradeoffs among communications performance, energy consumption, and complexity require improvements in future designs, for which we identify specific challenges and open research areas for MAC protocols deployed with next low-cost nanosatellite IoT systems.

## 1. Introduction

**Figure 1:**
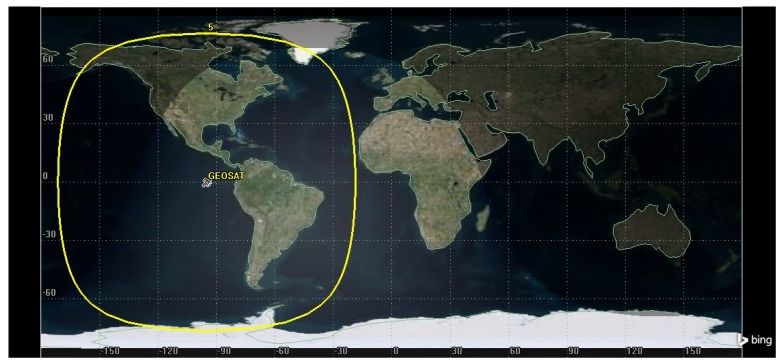
Approximate coverage of a geostationary satellite located at $91^{\circ }$ W.

From the beginnings of space exploration, satellites were large objects that took years to construct and cost billions of dollars for a single unit. With more advanced and smaller technologies, cheaper spacecraft (stand alone satellites and constellations of satellites) have evolved for diverse applications, telecommunication applications being most prominent. Commercial satellite companies like Iridium, Intelsat, O3b, and others offer a portfolio of products, including voice services, broadband, and sensor data collection, with extensive coverage of the Earth’s surface. For example, Figure 1 shows the approximate coverage of just one geostationary satellite located at a longitude of $91^{\circ }$ W.

With the internet of things (IoT), the paradigm that promises to revolutionize our world with the collection of enormous quantities of data, the connectivity demands are being increased around the globe. It is estimated that the IoT communications market will have an impact in the economy close to three to 11 trillion dollars per year in 2025 [1]. Nonetheless, terrestrial technologies do not fully cover the Earth’s surface yet. It is in such a scenario that satellite technology seems to offer the critical solution to the problem of global connectivity. However, traditional satellites are expensive—Iridium’s NEXT constellation of 75 satellites costs three billion dollars [2]—and thus novel, cheaper satellite solutions have become the focus of growing interest.

With the need for more coverage of the IoT networks and the search for cheaper solutions, nanosatellites may be the best answer for the global connectivity that the IoT demands. The nanosatellite standard, the CubeSat with a volume of less than one liter and a weight of less than one kilogram, also offers access to space and satellite development for countries that previously had no experience in space sciences. Nevertheless, the performance of such a solution will largely depend on the low-level protocols selected for the network architecture.

At the core of network architecture are the medium access control (MAC) protocols. Given the broadcast nature of channels in satellite communications, a MAC protocol ensures the proper coordination of frame transmissions, together with the logic for retransmissions and the recovery of data in case of collisions. In the past, there have been comprehensive reviews related to MAC protocols for satellite technology and also in the context of IoT. Peyravi [3] compiled a thorough revision and evaluation of MAC protocols for satellite communications. Although the study includes an evaluation with objective metrics such as throughput, buffer occupancy, scalability, stability, and reconfigurability, these metrics have been defined in the context of a constellation of geostationary satellites, which highly differ from the network conditions provided by smaller satellites deployed in lower orbits. Other similar surveys that focus on resource allocation and MAC protocol comparisons in conventional satellite systems are presented in Gaudenzi et al. [4] and Herrero et al. [5].

A more recent survey by De Sanctis et al. [6] makes the case of applicability of satellite communications for IoT and machine to machine (M2M) systems, also mentioning the potential of employing CubeSats within this context. Although the authors do provide a review of MAC protocols, no quantitative or comparative evaluation is provided for the MAC protocols reviewed in the work. Other works discussing the applicability of (small/nano) satellites in broadband internet access, IoT, and M2M communications can be found in [6,7,8,9,10]. The mentioned works, however, do not cover specific evaluations related to MAC protocols. Reviews devoted to the IoT, the enabling technologies, and services are also found in [11,12,13,14]. The focus of such works is more general except for the revision of MAC protocols for IoT presented by Oliveira et al. [14]; nevertheless, besides the fact that the mentioned works do not include a quantitative performance evaluation and comparison, a good part only discuss terrestrial IoT wireless technologies; hence, the discussion is oriented to different channel and network conditions from the ones addressed in this survey.

The contributions of this paper are threefold: (1) we review MAC protocols employed or proposed for satellite systems from a novel viewpoint that considers the restricted characteristics of CubeSat technology for wireless communications together with the particular requirements of IoT services and applications; (2) we provide a comparative quantitative and qualitative evaluation of the current protocols with metrics including communications performance (i.e., throughput, channel load, packet loss), dependency of network topology, implementation complexity, and energy consumption; and (3) we discuss the open research and implementation challenges to address by the next generation of nanosatellite networks for IoT environments.

The remainder of this paper is organized into seven sections. In Section 2 we present the fundamental aspects of satellites, space communication systems, and nanosatellites technology. It also includes a discussion about the IoT requirements. In Section 3 we introduce the specifics about the proposed IoT scenario served by a constellation of CubeSats. In Section 4 we provide the backgrounds on MAC layer protocols and introduce the metrics for evaluation. Section 5 presents a detailed review of MAC protocols designed and developed for satellite systems and other IoT-related technologies. It also includes a performance evaluation with objective metrics relevant to the IoT study scenario. In Section 6 we discuss the advantages and shortcomings of the existent protocols and identify the open challenges. Section 7 presents the final remarks.

## 2. Overview of Space and Communications Systems

### 2.1. Satellites Evolution

In the 1950s, the Soviet Union launched Sputnik I, the first artificial satellite that orbited the Earth. This milestone marked the beginning of a competition between two powerful countries that had, as one of its consequences, an accelerated technological development in aerospace sciences. Satellites created in the decades after the beginning of this competition were designed for very specific missions, and each development had its own subsystems—energy, command and data handling, attitude control, etc.—which allowed the particular requirements of a given project to be met. Such a design methodology involved an extremely expensive process due to the constant iterations necessary to create a new device, and the difficulties in reusing previous versions and designs.

One early shift occurred when, due to the use of modular systems, the main bus was designed to be flexible and reconfigurable according to the goal of each mission. As a result of reducing the costs in developing one unit, constellations of these spacecraft began to be feasibly designed and used by those countries and companies that could afford the still enormous cost of development. Depending on the configuration, these formations could increase the instantaneous global coverage and reducing revisiting times, among others benefits.

A large number of satellites now dot the skies for diverse applications such as navigation, imaging, meteorology, and communications. Some of the most significant applications are the following:

- Positioning systems: constellation of satellites located in medium height orbits (approximately 20,000 km) that make it possible to determine the position of an object on the Earth’s surface in a given coordinate system. There are several systems belonging to different countries, namely: GPS (USA—24 satellites), global orbiting navigation satellite system (GLONASS) (Russia—24 operational satellites), GALILEO (European Union—24 satellites), BEIDOU (China—17 operational satellites).
- Earth observation: Several satellites with a wide variety of cameras in different spectral bands have been sent to space. Defense and security, cartography, and meteorology are some of the disciplines that have benefited from these types of missions.
- Communications: Satellite systems that provide voice services, satellite television service, and narrowband/broadband connectivity through standalone satellites or constellations.

**Figure 2:**
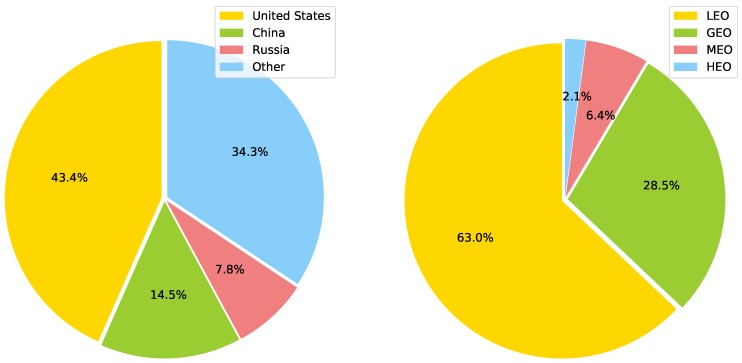
Active Earth orbiting satellites, separated by country of origin and type of orbit, from a total of 1957 active satellites reported until 30th November 2018. Data published by the Union of Concerned Scientists (UCS) in its annual report [15].

The Union of Concerned Scientists (UCS) maintains a count of operational satellites orbiting the Earth. In Figure 2 we illustrate the distribution, according to the country of origin and the type of orbit, for the 1957 active satellites reported up to 30th November 2018 [15].

### 2.2. Communication Satellite Systems

One of the areas in which satellites have been relevant is in communication networks. Due to the innate capacity of these spacecraft to cover the whole terrestrial surface, satellite systems are able to provide connectivity to remote or isolated areas that by other means are almost impossible to connect.

There are three main types of architecture used in satellite communication systems: store and forward, bent-pipe, and crosslink [8]. In the first, the satellite retrieves data from one point, stores it for some time, and then downloads it to the first ground station it establishes a connection with. In the second case, the satellite acts as a relay, collecting data and retransmitting it to another point on Earth. In the crosslink architecture, the data is transmitted immediately through a satellite network via inter-satellite links.

Satellite communication systems can be deployed in different orbits, offering a different set of services according to the channel/network conditions derived from the characteristics of the orbit of deployment. The types of orbits are the following:

- Geosynchronous equatorial orbit (GEO): This corresponds to an orbit whose rotation period is the same as the Earth’s. Consequently, the satellite seems to “stand still” to an observer at one point on the planet. To achieve this effect, the satellites are placed at a distance of approximately 35,786 km from Earth. Given such a long distance, the communication delays are considerable, in the order of 120 ms, in the satellite-ground direction or vice versa, for the best scenario; also, the transmission power required to establish effective links is high. Nevertheless, these systems have an excellent and broad coverage, reaching a 30% of the Earth’s surface. The placement process of a satellite into this orbit is an expensive task, and in order to remain at that position, the crew on the ground must perform orbital maneuvers from time to time.
- Low Earth orbit (LEO): Most of the satellites in space today are placed in this type of orbit. Its height ranges from 300–2000 km and, therefore, the delay in communications is low, in the order of tens of milliseconds for the worst case. The transmission power required to establish the links from this orbit are as low as hundreds of milliwatts [16]. Satellites in this orbit have low temporal and spatial coverage. Because of the speed—about 7.5 km per second for a satellite in a 500 km orbit—the Doppler effect has to be considered in these systems.
- Medium Earth orbit (MEO): Heights are between the low and geostationary orbits—2000–35,786 km. One example system, the O3b network, is placed at a height of 8000 km and has a theoretical minimum delay of 26 ms satellite-ground, or vice versa. All global navigation satellite systems (GNSS) constellations are placed in this orbit.
- Highly elliptical orbit (HEO): Orbits with a large apogee and a small perigee. The most famous of this kind is the Molniya orbit, which offers large coverage for high latitudes. Another example is the Tundra orbit. In Molniya, the apogee is greater than a geostationary—about 40,000 km. Satellites in this particular orbit have an approximate period of 12 h. The Soviet Union was the first country to use it to provide communication services throughout its territory and also to obtain meteorological images.

The classical services provided by satellite communication systems are the following:

- Broadband communications: The commercial satellite networks providing this service offer connectivity with broadband data rates. For example, the new Iridium’s NEXT constellation offers connectivity at 1.5 Mbps [17], whereas the Inmarsat’s BGAN HDR offers connections at 800 kbps [18]. Generally, stations on the ground require a large antenna along with a high transmission power to establish effective links. Satellites serving broadband communications usually operate in the Ka, Ku, L, and C bands.
- Voice services: Using small devices such as satellite telephones, these satellite systems offer voice connectivity on almost any part of the planet.
- Signaling services: In this area, some of the highlight services are the reception of automatic identification system (AIS) and automatic dependent surveillance broadcast (ADS-B) signals, which can track the path of vessels and aircraft, respectively.
- Sensor data collection: These satellite systems offer services at low data transfer rates, which allow data to be retrieved from small sensors placed on the ground.

Table 1 provides a list of some of the commercial constellations providing communication services in different orbits.

### 2.3. CubeSats

Traditionally, most of the projects for designing and building satellites have been excessively expensive. They involved complex designs and, consequently, long development time spans. However, starting in the 1980s, a new paradigm was established that significantly reduced the size of some satellites, leading to the appearance of microsatellites and, in the 2000s, the creation of nanosatellites or CubeSats: aircraft whose weight is equal to or less than one kilogram.

**Figure 3:**
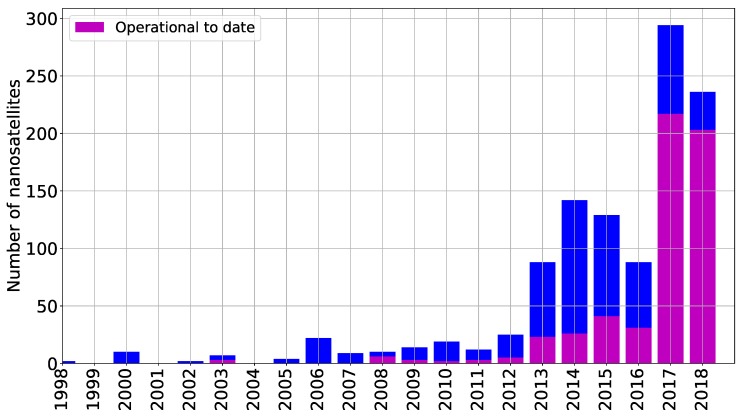
CubeSats launched since 1998. Data taken from the database at [19].

The CubeSat standard was created in 1999 at the California Polytechnic State University in conjunction with the Stanford University’s Space Systems Development Lab. The development of this standard aimed at improving access to space by providing opportunities for satellite development, design, and construction to institutions that could not do so with the classical paradigm. Figure 3 shows the number of Cubesats launched and operational to date.

The basic design of a CubeSat consists of a 10-cm cube—called 1U—which must contain the primary subsystems for the operation: an onboard computer, batteries, transmitters and receivers for communication, and attitude determination and control system (ADCS), among others. The cubic shape and volume defined for this new standard considerably reduce launching costs, but, at the same time, incorporate restrictions regarding availability of computational resources, energy, and volume, among others.

Initially, the development of nanosatellites was intended to test components and study their behavior in the space environment. Nowadays, applications have spread widely with projects led by universities, governments, and commercial entities. Moreover, and of interest to the authors, this technology represents an excellent opportunity for developing countries to exploit space resources in addition to providing a tool to democratize the use of space [20].

In South America, for example, several countries have taken advantage of nanosatellite technology to promote educational initiatives within universities, including, the Libertad-1 in Colombia (Sergio Arboleda University) [21], PUCPSAT in Peru (Catholic University of Peru) [22], SUCHAI-1 in Chile (University of Chile) [20], to mention just a few. In the commercial field, new companies have appeared in the market for developing and selling CubeSat parts and pieces; other companies make use of CubeSats for applications such as satellite imaging collection. Government agencies, such as NASA and ESA, developed nanosatellite-related missions. One of the most notable examples is the experimental use of two CubeSats—Mars Cube One (MarCO) A and B—as communication relays for the InSight-1 probe that landed on Mars in November 2018.

### 2.4. IoT and M2M Requirements

Cisco forecasts that by the year 2020 the number of devices connected to the internet will exceed 50 billion [23], an increase that raises a connectivity challenge for these new massive networks. It is in this field that the capabilities of the new low-cost nanosatellite networks could be instrumental in achieving a global connectivity, as demanded by the fifth generation networks.

The IoT and machine-to-machine networks are characterized by their intention to meet one or more of the following requirements:

- Efficient performance against explosive traffics
- Low data rates in terminals
- Energy efficiency
- Low cost terminals
- Mobility and scalability
- Minimization in the use of spectrum
- Minimum signaling
- Data security
- Data integrity
- Reliability
- Robustness
- Flexibility

**Figure 4:**
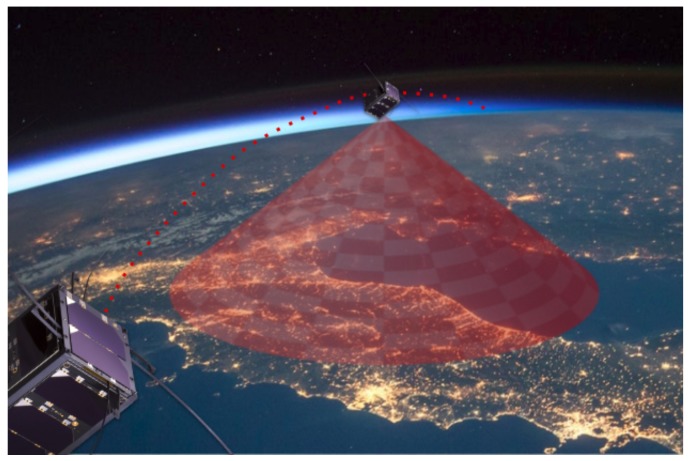
Illustrative scenario in which a CubeSat (or a constellation of them) provides connectivity for internet of things (IoT) applications.

In the case of terrestrial wireless access networks, various solutions have been developed to meet the above requirements. For low consumption sensor arrays deployed across extensive areas, technologies such as LoRaWAN [24] and Sigfox [25] are available; for sensors and actuators networks deployed in urban environments, there are Wi-Sun [26] and NB-IoT [27], to mention some of the available technologies. There are also autonomous sensor networks interconnected to provide solutions to specific applications involving short-range technologies, such as IEEE 802.15.4 [28]. Despite the advances with the introduction of these new technologies, there are scenarios for which existing networks do not offer feasible solutions. Remote places without connectivity still exist; also, some monitoring applications in isolated places require devices with high and unpredictable mobility to collect information on-the-move (e.g., monitoring of wild animals in areas of difficult access). Considering the scenarios mentioned above, microsatellites and CubeSats appear as viable alternatives to cover the gap in providing fully connected global communications networks for the IoT [6,10]. An example scenario of a CubeSat providing IoT connectivity is illustrated in Figure 4.

One of the challenges of these new massive networks is to enable the many terminals to share a physical resource—the broadcast communications channel—in an efficient and orderly manner. Such a challenge would necessarily make use of the medium access control layer, which corresponds to a sub-layer of the link layer of the open system interconnection (OSI) model and is responsible for coordinating frame transmissions in broadcast links. The specific MAC protocol used for IoT applications will need to fulfil a number of requirements including increased average throughput, to meet a minimum level of fairness as well as to comply with the resources, requirements, and limitations of the access technology in use. Another critical aspect to consider in the choice of a MAC protocol is the network topology and how much knowledge the nodes have or need about that topology.

To examine the fulfillment of the IoT and M2M networks requirements, from a MAC layer perspective in the case of this study, together with the restrictions imposed by the capabilities of the CubeSats, will shed light about the viability to provide IoT connectivity using nanosatellites. The reviews and discussion presented in the coming sections address all of the IoT and M2M requirements listed above, except the ones related to data security and data integrity. Whilst security aspects are of paramount importance in the IoT ecosystem, we direct the interested reader to specialized works on this subject discussing security threats and mitigations for a variety of IoT technologies and architectures [29,30,31,32] and specifically for satellite communications [33,34,35].

## 3. IoT Scenario of Study

In order to exploit the full capabilities of the IoT, connectivity is a major issue to be solved in the task of recovering the amount of data generated by the—expected—billions of sensors forecasted to be deployed in coming years. Although some existent IoT technologies, like LoRa and SigFox, claim to have large coverage—40 and 20 km in rural environments, respectively [36]—they are not even close to the coverage that satellite systems can provide. Nevertheless, satellite connectivity is still considered very expensive and poor in terms of energy efficiency. It is in this context that researchers consider that the CubeSat standard could be a feasible solution to mitigate the above mentioned disadvantages of traditional satellite networks, lowering the costs of satellite systems and making it a viable alternative to current wireless technologies for IoT connectivity.

In this context, the scenario to be considered in this review corresponds to a CubeSat constellation, with no inter-satellite connectivity, whose main purpose is retrieving small amounts of data from sensors placed on the ground at a low data rate. The constellation will be deployed in several orbital planes belonging to the Low Earth orbit; each orbit with a height ranging between 500–600 km and with an inclination close to $97^{\circ }$. Each nanosatellite from the constellation will face the same problem: as it orbits around the planet, it will have to recover data from a network on Earth whose number of nodes and geographic distribution is unknown and (possibly) changing continuously. Analyzing the case for one satellite—the master—and several ground sensors—the slaves—will be representative of the problem to be faced by the complete fleet.

The satellite communication system uses the 400 MHz band, which has low propagation losses compared with the typical bands employed by commercial companies offering satellite broadband services. Such frequencies are in the range of the amateur frequency band used and proven to work by most of the CubeSat projects deployed to date [16]. The communications are half-duplex and have an expected maximum data transmission rate of 100 kbps, which is similar to the rate offered by commercial developments of transceivers for nanosatellites [37]. It is assumed that the antennas in use, as well as the transmission power and the receivers’ sensitivity, are adequate to establish effective data links for most nodes under the coverage area of the nanosatellite. However, it is expected that the furthest nodes from the nanosatellite are less likely to generate a correct link due to the greater distances to be covered.

As mentioned above, the sensors are distributed randomly in any geographical area on Earth. A sensor node is not aware of the network topology, and the spacecraft does not know in advance how many devices needs to serve in an area of coverage. Each sensor generates a quantity of data independent of the others. It is also assumed there is no temporal synchronization among the sensors nodes, nor between the sensor nodes and the nanosatellite.

## 4. Background on MAC Protocols

MAC layer groups a set of protocols and mechanisms in order to distribute the resources for the nodes to make an effective (and efficient) use of the communications channel. The resources are typically distributed in terms of time assignment, frequency assignment or code assignment. In the particular case of broadcast links, a MAC protocol is in charge of coordinating the frame transmission.

Each MAC protocol is designed to cover different requirements, and its performance can be quantified with different metrics. In some cases, the priority is set to the performance concerning data transmission rate, for which the normalized offered load and the normalized throughput are measured. Other priorities may include measurements of delays in sending data or the packet loss ratio (PLR). In the particular case of IoT applications, there may be limitations regarding processing capabilities, available storage, hardware complexity, and energy consumption.

In this section, the authors provide a set of metrics that can quantify the fulfillment of the different requirements objectively. We also present the traditional categorization employed to classify the existent MAC protocols for broadcast channels.

### 4.1. Evaluation Metrics

#### 4.1.1. Normalized Offered Load (C)

The normalized offered load (C) is the quotient between all the data injected into the network and the maximum data that could be sent at the transmission rate of the link. The latter corresponds to the product of the transmission rate and the total transmission time. The normalized offered load is calculated according to the following formula:

(1) $$C=\frac{\sum D_{i}}{T_{x}\cdot t_{t}},$$

where $D_{i}$ is the data sent to the satellite by sensor *i*, $T_{x}$ is the link transmission rate, and $t_{t}$ is the total transmission time.

#### 4.1.2. Normalized Throughput (S)

The normalized throughput is the quotient between the data received by the satellite in a given time and all the data that could be sent continuously at the transmission rate of the link. It can be interpreted as how effective is the use of the channel. It is always true that S≤C. The normalized throughput is calculated according to the following equation:

(2) $$S=\frac{D_{r}}{T_{x}\cdot t},$$

where $D_{r}$ is the amount of data received by the satellite, $T_{x}$ is the link transmission rate, and *t* is an arbitrary time.

#### 4.1.3. Packet Loss Ratio (PLR)

PLR corresponds to the proportion of data lost or received with errors due to miscoordinations of frame transmissions, and that cannot be recovered over the total amount of data sent. The PLR is calculated as follows:

(3) $$PLR=\frac{P_{l}}{P_{s}},$$

where $P_{l}$ is the number of lost packets and $P_{s}$ is the number of packets sent. This ratio turns out to be important when energy efficiency is required, since a high PLR may trigger a high number of retransmissions when implementing a reliable link layer, which may mean more waste of energy. In general, the channel performance is analyzed by examining the supported channel load for a target PLR, which is commonly considered on the order of $10^{-3}$ in the literature. In some cases, the normalized load achieved with a target $PLR=10^{-3}$ is very low, making it necessary to consider worse PLR values in the analysis, e.g., $PLR=10^{-2}$.

The relation among the three metrics presented above is described by the following equation:

(4) S=C(1−PLR).

#### 4.1.4. Energy Consumption

From the point of view of MAC protocols, energy consumption is directly affected by the length of time in which data is being sent and received; to a lesser degree, energy consumption is also affected by the amount of processing required by the protocol. To evaluate the energy consumption, the length of time the transceiver is in transmission, reception, and idle modes should be compared. The consumption on each state depends specifically on the model of transmitter/receiver that is being used and the chosen MAC protocol. For example, in SigFox, the current consumption is 11 mA in reception mode and 125 mA in transmission mode [38]. The peak current consumption is about 32 mA and a range from 120 mA to 300 mA, in the cases of LoRa and NB-IoT, respectively [39].

In general scenarios, the main energy limitation is found in the terminal nodes, since in most cases the receiving station has a virtually infinite energy source (e.g., a base station in a cellular network, a WiFi access point, etc.). In our study scenario, the case is different since CubeSats may also have energy limitations. Nevertheless, it is expected that energy limitation in the sensor nodes will be considerably higher than in the spacecraft.

#### 4.1.5. Complexity of Implementation

In the context of CubeSats and low-cost satellite solutions, the complexity of implementation turns out to be a relevant factor. For this reason, aspects such as the need of high processing availability, the presence of very specialized hardware, and large amounts of required storage, should be considered as directly impacting the complexity of a given MAC protocol.

Usually, on-board computers (OBC) employed on CubeSats are microcontrollers such as the Microchip PIC24 or the Texas Instruments MSP430, which are very limited in terms of computational resources. Newer OBCs using the ARM Cortex family or ATMEL devices are already available in the market for nanosatellites, but they still are in the category of modest processors.

### 4.2. MAC Protocols Categories

A brief categorization of the MAC protocols is provided as follows [3]:

- **Fixed Assignment**: Protocols in this category are characterized by assigning a limited resource equitably and fixedly between different interlocutors. The resource can be a frequency channel, a time interval or a code, deriving in the well-known mechanisms frequency division multiple access (FDMA), time division multiple access (TDMA), and code division multiple access (CDMA). These protocols are characterized by being easy to implement, as well as being efficient in link usage when they occupy all or most of their resources. However, protocols following a fixed assignment are not very flexible to changes in data rates, nor are they tolerant to variations in the number of stations since they require a coordinated allocation among all the stations involved.
- **Random Access**: These protocols are characterized by having a non-fixed number of users that, without prior coordination, make use of the same channel (i.e., contention-based protocols). Since the allocation of resources is random, more than one device may win the right to use the channel at the same time, causing frame collisions. Therefore, protocols in this category cannot guarantee the successful arrival of frames. Depending on the scenario, these protocols may waste system capacity in failed transmissions (and retransmissions). However, they have a fundamental role in networks whose previous characteristics (number of nodes, nature of traffic, etc.) are not known in advance.
- **On-demand**: These protocols are designed for scenarios in which the terminals require sending an unequal and variable amount of data; in that case, on-demand protocols can vary the allocation of resources depending on the nodes requirements. For example, a TDMA-based protocol may assign additional time-slots to nodes with higher requirements regarding data rate. To manage the variable assignment of resources, these protocols usually require extra control signaling, such as the incorporation of the packet generation rate of each terminal as additional control information in every message.
- **Adaptive**: These correspond to protocols designed to manage variable network conditions. These protocols are intended to change the MAC logic dynamically. For example, when communication is carried out among a few terminals, the MAC employs a random access scheme; conversely, when the number of devices increases, it uses a fixed allocation scheme.
- **Reservation**: The goal of these protocols is to achieve a collision-free allocation of resources. A typical way to achieve the collision-free scheme is the use of a subchannel dedicated to the coordination of access for each station, in such a way that only one station transmits at a given time. In that subchannel, the devices may rotate a testimony (i.e., a token) that indicates who has the right to transmit on the channel. Most of these protocols make use of TDMA or variations of Aloha to assign the token.

The last three categories are, in a general way, hybrids of the first two. This is mainly because the network characteristics—number of nodes, data generation rates, network explosiveness, etc.—have a nature that is essentially either random or deterministic. In this way, the dominant categories that match the network characteristics are either random access or fixed assignment protocols.

In this work, the MAC protocols selected for review corresponded mainly to random access and its derivations. These schemes were selected because, in the study scenario, it is infeasible to predict the state of channel congestion at all times, which and how many nodes are within nanosatellite coverage, and the amount of data each node wants to transmit.

## 5. MAC Protocols for Satellite IoT and M2M

The early satellite solutions traditionally employed protocols mainly based on fixed assignment (e.g., CDMA, FDMA, and TDMA). In some cases, the protocol was combined with a random access scheme to perform the adaptive assignment according to the demand of the nodes. An example of an early protocol is the demand assignment multiple access [40]. Nonetheless, as mentioned before, considering the nature of our study scenario, protocols in the random access category are more relevant and suitable for the comparative evaluation.

The protocols selected in this section included several descendants of the well-known Aloha protocol, since such derivations are present in current satellite systems and modern IoT technologies such as LoRa and Sigfox. The selection also included other significant—and more modern—random access protocols that were considered suitable for the IoT scenario described in Section 3, all applicable to satellite environments and other IoT technologies. Such protocols make use of advanced techniques like interference cancellation, and adaptiveness, among others.

### 5.1. Aloha-Based Protocols

The most representative random access protocol—and the inspiration for many other MAC protocols—is Aloha, developed in 1970. Although this protocol is quite old and simple, in current IoT developments Aloha plays a fundamental role. For example, leading IoT technologies that use variations of this protocol are LoRa and SigFox. Furthermore, there are several applications and modifications to Aloha reported for satellite environments in the literature. Some of them can be found in [41,42,43,44] for the interested reader.

In Pure Aloha, nodes send data when they have data to send, hoping that a collision does not occur. When the reception of a packet is successful, the receiver sends an acknowledgement (ACK); otherwise, nodes retransmit the same packet after a random time [45,46]. The performance of this protocol is quite modest. It achieves a maximum normalized throughput of S=0.18 when C=0.5. In terms of packet loss rate, it achieves a $PLR=10^{-3}$ for an extremely low normalized load of $C<10^{-3}$. The advantage of this protocol lies in its simplicity of implementation, since it does not require pre-coordination or extra access control signaling [47]. When there is low load in the channel, the energy consumption of Aloha is efficient, since it only requires sending data and the reception of an ACK, so the active consumption due to transmission and reception is proportional to the data and ACKs transmission delays. Nevertheless, for high channel loads, the packet losses due to collisions become high, which causes more retransmissions, overloading the channel with the associated wasting of energy.

#### 5.1.1. Slotted Aloha (S-Aloha)

The most similar version to Aloha is S-Aloha. It consists of discretizing the channel, where each time slot has the duration of a packet transmission time [46]. The purpose of discretizing is to avoid partial collisions among packets. S-Aloha is used, for example, in the sending of short packets and requests to initiate communications in the DVB-RCS standard [48].

S-Aloha achieves a low normalized throughput of S=0.368 when the normalized load is C=1. Similar to the case of pure Aloha, a channel load of $C<10^{-3}$ is supported when the $PLR=10^{-3}$. In the case of a higher packet loss, $PLR=10^{-2}$, the normalized load is increased to C≃0.01. Regarding the complexity of implementation, it can be said that S-Aloha adds additional complexity since it requires all the nodes to be synchronized, both on the ground and also on the satellite. Such a synchronization requires us to consider the time margins in order to align the time-slots among nodes that have different delays. Similar to pure Aloha, this protocol proves to be quite inefficient for high channel loads due to the need for retransmissions.

For IoT aplications, S-Aloha is a good option in scenarios where the offered load is low and the delays between nodes and base station do not have a large variation, but it becomes impractical if the delays imposed on a satellite link are considered. An application of this protocol on the recent Weightless-N IoT technology is reported in [49].

#### 5.1.2. Diversity Aloha/Slotted Aloha (DA/DSA)

This protocol is considered for systems that have large transmission delays (e.g., satellites in GEO orbits) and for which confirmations of packet reception are impractical [50]. In the diversity Aloha/slotted Aloha (DA/DSA) protocol each terminal sends two or more copies of a packet at different randomly selected times, without waiting for the reception of an ACK. The idea is to increase the probability of packet reception and to avoid retransmissions; however, the consequence is an overloaded channel. An application of this protocol in satellite environments is observed in the IP-over-Satellite system for sending short packets and registration [51,52].

The maximum performance of this protocol is reported to be lower than for slotted Aloha. In DSA, a maximum normalized throughput of S≃0.3 is achieved for a C≃0.6. However, when C<0.5, the performance of DSA is slightly better. In the case of a $PLR=10^{-2}$, the protocol supports a normalized load of C≃0.05 (compared to a C≃0.01 in S-Aloha). DSA is similar in behavior to S-Aloha in terms of implementation complexity and energy efficiency.

DA/DSA is suitable for links with large delays and offered loads less than C=0.5. Nonetheless, in IoT scenarios the channel load will tend to increase progressively with time, consequently DA/DSA may not meet the scalability requirement, a crucial aspect in an IoT system like the one described in the study scenario.

#### 5.1.3. Spread Spectrum Aloha (SS-Aloha)

**Figure 5:**
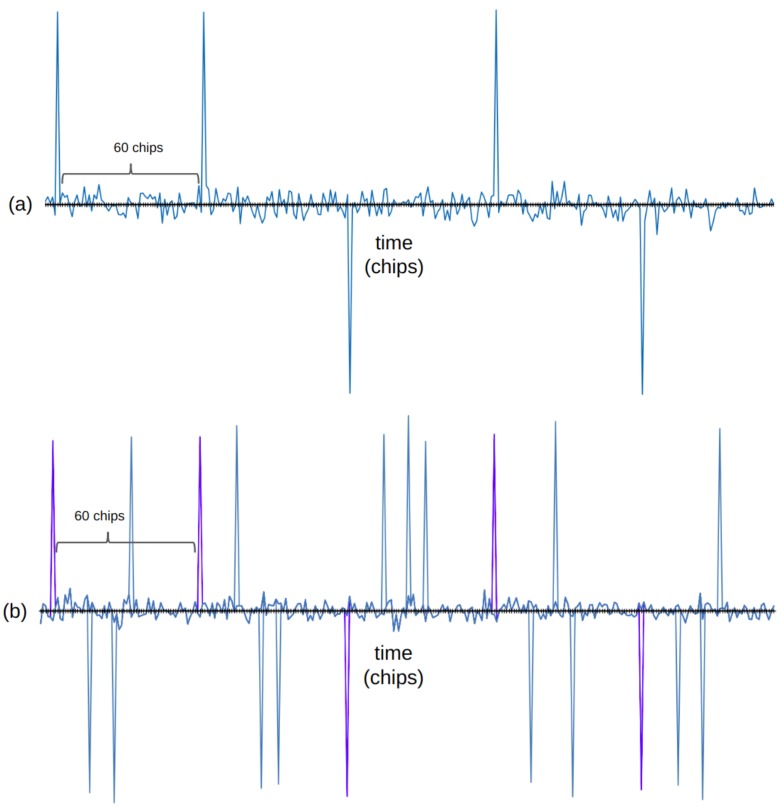
In (**a**), the signal received by the satellite from one transmitter. In (**b**), the signal received by the satellite from four transmitters; signals are still decodable because of the offset of chips between the different terminals. Figure adapted from [55].

The protocol is proposed to provide random multiple access over an unsynchronized channel. SS-Aloha uses spread spectrum techniques to send messages; it is similar to a CDMA protocol where each terminal uses the same code to spread the signal and accesses the channel without coordination (like in Aloha) [53]. The multiple access capability is given by the large bandwidth employed instead of the assignment of different codes. In Figure 5, we illustrate the responses of a correlator detector (at the satellite’s receiver) applied to signals from one terminal (see Figure 5a) and four terminals (see Figure 5b). In this example, a spreading factor SF=60 is used, in consequence, 60 chips are placed between two consecutive bits from the same terminal. In order to achieve multiple access, spread spectrum Aloha (SSA) makes use of the offset in chips between two signals, so in Figure 5b, the messages from the four terminals are still decodable. Previous evaluations of the SS-Aloha protocol in a satellite environment are reported in [54].

Regarding the performance of SS-Aloha, thanks to the use of proper error correction codes, the protocol achieves a maximum normalized throughput close to S≃0.6 for a given load of C≃0.7. In the case of $PLR=10^{-3}$, the system supports a load of C≃0.5 [5]. This protocol has a reduced level of complexity because it does not require synchronization. However, the spread spectrum technique has a strong dependency on the signal to noise plus interference ratio (SNIR) threshold in the demodulator to operate correctly. SS-Aloha shows to be efficient in terms of energy consumption; similar to Aloha, SS-Aloha only requires the sending of data packets with no need to send extra control signaling or synchronization information.

The simplicity required in transmitters in addition to the good performance of SS-Aloha, compared to previous protocols, may make this protocol suitable for IoT scenarios. However, when employed in LEO satellite links, where the expected power imbalance is high, the performance of this protocol drops drastically, behaving similarly to S-Aloha.

#### 5.1.4. Enhanced Aloha (E-Aloha)

When sensor nodes of a telemetry application transmit data readings, they are usually configured to send packets in a periodical way, with a fixed time interval between messages. In this scenario, the E-Aloha protocol has been proposed as a simplified version of Aloha. At the time of sending, nodes simply initiate transmission, with no additional control to avoid collisions; a confirmation of reception is also not considered in the protocol. For some applications, nodes may end up with the same time interval between packets, in which case the nodes will collide permanently, with no effective communication. To avoid this situation, E-Aloha defines a time window—considerably longer than the packet transmission time—located around the fixed sending time. Within this window, each node selects randomly a new sending time, thus reducing the chances of permanent collisions among nodes using the same time interval.

**Figure 6:**
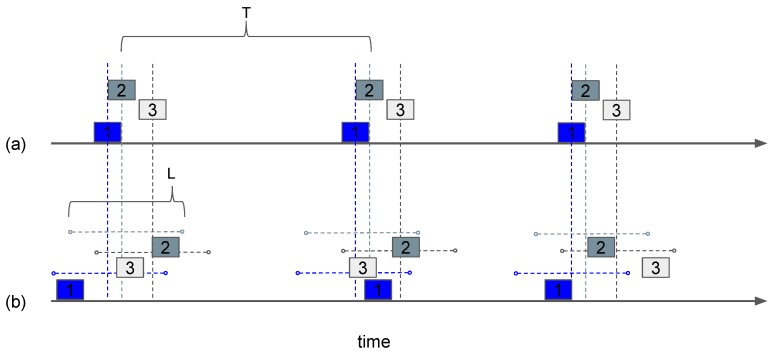
Example of E-Aloha operation where three users send packets at a rate of $\frac{1}{T}$ packets/s. In (**a**), the users wait a fixed time (*T*), in consequence, there is a permanent collision between the users 1 and 2; whereas in (**b**) there is a time window (*L*) for selecting a new random sending time in order to avoid permanent collisions. Figure adapted from [43].

Figure 6 illustrates the behavior of the protocol for nodes originally colliding over the same time interval (see Figure 6a), and the corrections made through the use of a time window around the fixed sending times (see Figure 6b). E-Aloha was introduced in [43] for use in satellite systems devoted to telemetry such as Argos.

The performance evaluation of this protocol considered the periodic traffic characteristic of typical telemetry systems [43]. The reported results indicate that for a $PLR=10^{-1}$, the protocol achieves a normalized throughput of S=0.091, considering a channel load C=0.101. The performance is, then, very similar to the one reported for S-Aloha (C=0.1 for a $PLR=10^{-1}$), with the advantage that E-Aloha does not require time synchronization. Furthermore, there is less complexity in implementing E-Aloha than for Aloha, in particular since E-Aloha does not require a specific logic implemented at the receiver. By not requiring ACK, this protocol is energy efficient when the load on the channel is very low (C∼0.1). For higher loads, the performance of the protocol drastically decreases due to the high number of collisions.

The simplicity of this protocol is desirable for the implementation on the IoT terminal node. It actually behaves well for the reported channel loads found in the systems where the protocol has been implemented. However, E-Aloha may lack the necessary scalability to support the future IoT networks.

#### 5.1.5. Random Frequency Time Division Multiple Access (RFTDMA)

**Figure 7:**
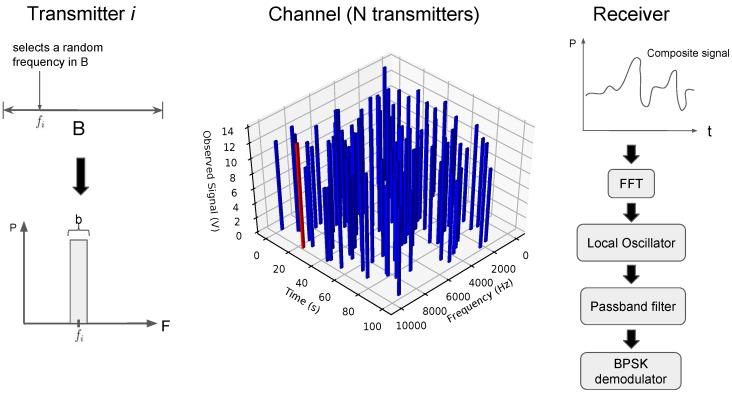
Random frequency time division multiple access (RFTDMA) communication process. Temporal and spectral random access from hundred of nodes.

Despite not being reported in satellite environments, this protocol has an important relevance in low power wide area (LPWA) technologies, more precisely for its use in SigFox. Considering the ultra narrowband (UNB) technology, this protocol acquires prominence when low cost transmitters that do not require expensive oscillators are required to perform a precise adjustment of the carrier frequency. Taking this into account, random frequency time division multiple access (RFTDMA) uses the time and frequency to send messages without discretization (as in pure Aloha) [38]. Figure 7 illustrates the process of a transmitter selecting a frequency according to the protocol, a representation of the channel resulting from many nodes transmitting at the same time and, finally, the receiver’s architecture to retrieve the data out of the composite signal.

The performance of this protocol is calculated in [56]. Its maximum normalized throughput is lower than 0.1 for C≃0.25, and the PLR results are not reported as a function of *C*.

### 5.2. Reservation and Adaptive Protocols

#### 5.2.1. Reservation Aloha (R-Aloha)

**Figure 8:**
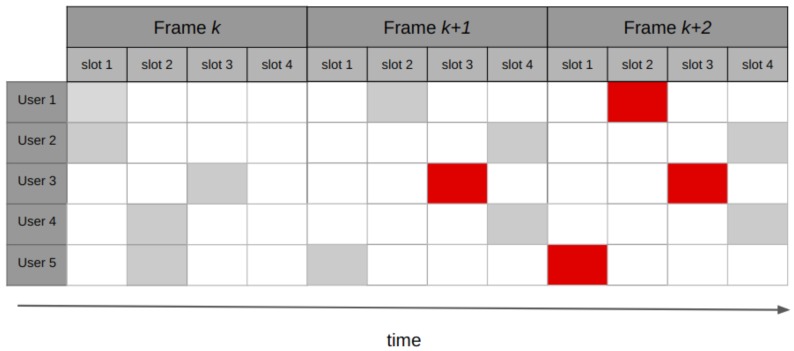
R-Aloha operation: five users contending for channel access. User three transmits successfully in slot three of frame *k*, and reserves the same slot in future frames k+1 and k+2. Users one and five achieve successful transmissions in frame k+1, and place their reservations in frame k+2 for slots two and one, respectively. Red packets correspond to active reservations.

This protocol divides the time into *m* slots, each with the duration of a packet transmission time. The slots are grouped in frames, and the nodes randomly choose one slot per frame to send a packet. If a node successfully sends a packet, it proceeds to reserve the same slot in future frames. At the end of each frame, the receiver responds with an ACK, also indicating what the available slots for the next cycle are. An example of the operation of reservation Aloha (R-Aloha) is shown in Figure 8. The R-Aloha protocol was proposed for incorporating satellite communications in the ARPA network [57].

The throughput of this protocol depends mainly on the number of packets sent by each node during a reservation. In the worst case, the performance of R-Aloha is similar to the S-Aloha. When the protocol holds reservations for a large number of frames, the normalized throughput *S* approaches to 1 [58].

Although R-Aloha reports having a normalized throughput that tends to 1, it may not meet the requirements of IoT scenarios: the protocol has a good performance when the nodes disputing the channel resemble the number of slots in each frame, and also when the reservations made by each terminal last a large number of frames. However, when the scenario does not comply with these assumptions, R-Aloha turns out to be an unscalable protocol. Moreover, for IoT applications where nodes typically have small and/or infrequent amounts of data to send, the reservation mechanism of R-Aloha may result impractical.

#### 5.2.2. Carrier Sense Multiple Access with Collision Avoidance (CSMA/CA) with RTS/CTS

**Figure 9:**
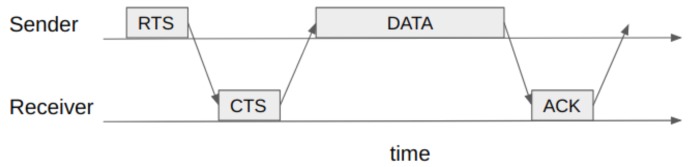
Exchange of packets for a successful channel reservation with request-to-send/clear-to-send (RTS/CTS) signaling in carrier sense multiple access with collision avoidance (CSMA/CA).

A classical protocol in wireless network, the carrier sense multiple access with collision avoidance (CSMA/CA) defines the monitoring of the channel before sending a message. If a node senses the channel is busy, it refrains from transmitting and enters an exponential backoff stage; otherwise, it issues a reservation request by means of a small broadcast message (i.e., the request-to-send (RTS) packet). When there are no collisions and the receiver decodes the RTS correctly, another broadcast message granting the reservation (i.e., the clear-to-send [CTS] packet) is sent by the receiver. Upon reception of the CTS, the sender proceeds to send the data packet [59]. Figure 9 illustrates the exchange of packets when a successful reservation is placed in CSMA/CA with RTS/CTS. The protocol has been evaluated in a LEO satellite environment considering different back-off distribution functions [60].

The maximum performance regarding throughput of this protocol varies between S≃0.5 and S≃0.8, since its performance depends on factors such as the packet length, number of nodes, and the number of hidden terminals, among others. As reported in [59], for an example network with 10 stations and no hidden terminals, the protocol achieves a normalized throughput of S=0.75 when the load is C=0.8. In the presence of hidden terminals—a common scenario for a satellite system with ground terminals distributed over a large area—if there is a 5% probability of hidden terminals for a total of 10 stations on ground, the normalized throughput is S=0.65. By increasing the number of stations to 50 in the same case, the normalized throughput falls to S=0.57. PLR values for this protocol are not reported since packet losses are avoided when using the reservation mechanism.

In terms of complexity, CSMA/CA is still a simple protocol: it does not impose high processing demands on the nodes beyond the ability to transmit and receive. Conversely, the power consumption of CSMA/CA is high, since each node must permanently listen and monitor the channel.

CSMA/CA is widely used in wireless IoT technologies, such as ZigBee, D7AP, and other short-range wireless sensor networks [49,61]. However, for the protocol to be implemented on a satellite IoT network, it may be impractical for two reasons: first, the probability of hidden nodes in a satellite scenario is high due to a wide geographic distribution area; and second, the long delays of the different devices on ground make the channel sensing ineffective even when there are no hidden terminals.

#### 5.2.3. Fixed Competitive TDMA (FC-TDMA)

**Figure 10:**
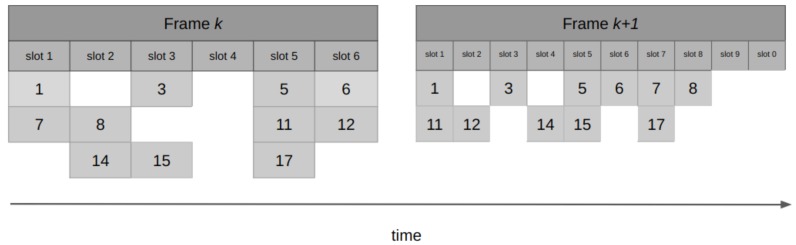
Fixed competitive TDMA (FC-TDMA) operation. In the first frame, packets collide in every slot. For the following frame, the number of slots is increased, resulting in five successful transmissions out of ten slots. The number in each packet represents the node’s ID.

Similar to R-Aloha, the fixed competitive TDMA (FC-TDMA) protocol defines a set of *m* slots grouped in frames. In each frame, the nodes select a slot for transmission in a pseudo-random manner [62]. To calculate the allocated slot for packet transmission, $n_{slot}$, a node employs its ID—a previously assigned integer number—and follows the calculation shown in (5). The receiver has to estimate the number of stations on the ground based on the colliding slots and those with successful transmissions; the frame is further divided according to that estimation. An example of the operation of FC-TDMA is illustrated in Figure 10. The authors in [62] have suggested this MAC protocol for LEO satellite systems.

(5) $$ID\%m=n_{slot}.$$

The theoretical maximum normalized throughput of the protocol is S=1 with a load of C=1. This performance corresponds to a scenario with a number of slots in the frame equal to the number of devices on the ground, and all the devices having an assigned ID such that there are no collisions. The complexity of this protocol is related to the TDMA functionality and the variable slot lengths. In addition, the algorithm for estimating the number of terminals on the ground may impact both the energy performance and the complexity. However, the details of this algorithm are not provided in the literature.

Similar to R-Aloha, the maximum normalized throughput of FC-TDMA is achieved under very specific conditions: (a) when the number of devices is constant; and (b) when there are no conflicting IDs among all users. However, FC-TDMA could potentially match the traffic characteristics of an IoT application as long as the estimation of the number of nodes under its coverage is accurate and fast.

### 5.3. Interference Cancellation-Based Protocols

#### 5.3.1. Contention Resolution Diversity Slotted Aloha (CRDSA)

As the name indicates, contention resolution diversity slotted Aloha (CRDSA) is based on the DSA protocol. In addition to sending two or more copies of a packet, CRDSA iteratively resolves the collisions that occur at the receiver through the use of a successive interference cancellation (SIC) mechanism [51]. For a given frame, nodes send two or three copies of the packet in different slots. The entire frame is then stored in a digital memory at the receiver. Furthermore, each packet includes control data that indicates in what slot the “twin” packet has been sent. In this way, when a packet is decoded correctly by the receiver, the latter retrieves the information regarding the arriving slot for the “twin” packet. With this information, the receiver is able to perform the interference cancellation method. The CRDSA protocol has been developed and included in the DVB-S2/RCS standard [63].

In terms of communication performance, when CRDSA uses two copies per packet, it achieves a maximum performance of S=0.52 for a given load of C=0.65. In the case of a $PLR=10^{-3}$, the supported channel load is C≃0.05, improving to C≃0.26 when the $PLR=10^{-2}$. Note that CRDSA achieves a high normalized throughput performance for a low PLR; consequently, the protocol is also considered energy efficient: although each node must send each packet two times, no further retransmissions are required. Nevertheless, protocols based on interference cancellation have a high dependency on a good channel estimation, which adds complexity to the implementation. In addition, CRDSA also reports high demands regarding processing and storage capabilities at the receiver, together with requiring synchronization among nodes.

**Figure 11:**
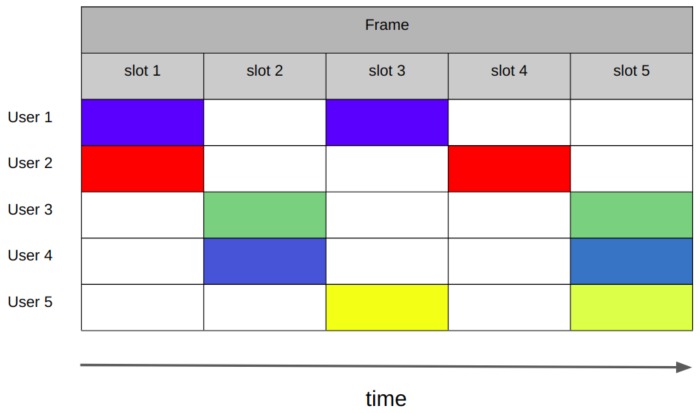
Example of the operation of contention resolution diversity slotted Aloha (CRDSA) over one frame. A collision-free packet from user two is received in the fourth slot. The packet includes the position of its “twin”, located in slot one. An interference cancellation method is then applied to the packet. In this example, packets from users one, two, and five can be successfully recovered, achieving an S=0.6 for a load of C=1.

To address the drawbacks reported for CRDSA, the protocol has evolved with adaptations such as multi-frequency CRDSA (MF-CRDSA) and spread spectrum CRDSA (SS-CRDSA) [64]. The former deals with the problem of requiring power peaks to send complete messages over small time slots. By dividing the available spectrum in multiple channels, the time slots in MF-CRDSA can be longer, thus avoiding very high power peaks but at the expense of the protocol’s performance. The latter protocol, SS-CRDSA, addresses the problem of “loops” in the original CRDSA. A loop occurs when two different sources send replicas of their packets over the same slot, making it impossible to apply a successful interference cancellation (see for example users three and four in Figure 11). To avoid the loop, SS-CRDSA uses spread spectrum techniques and randomly associates codes to each packet.

#### 5.3.2. Irregular Repetition Slotted Aloha (IRSA)

This protocol represents an improvement over CRDSA. Although it also requires the nodes to send copies of packets in randomly chosen slots, the difference lies in that the number of duplicates for each packet varies depending on an optimized distribution probability. The protocol is intended to improve the performance over uplink satellite channels [65].

Irregular repetition slotted Aloha (IRSA) achieves a maximum performance of S=0.8 for a given load of C=0.85. In the case of a $PLR=10^{-3}$, the supported normalized offered load rises to C≃0.7. The complexity of this protocol is similar to the complexity of CRDSA, but it adds the difficulty of calculating the different number of repetitions per packet. Given its performance, IRSA is considered an energy efficient protocol for normalized offered loads near C=0.7, because of its low PLR.

#### 5.3.3. Coded Slotted Aloha (CSA)

A protocol inspired by IRSA and CRDSA in which the nodes divide packets before transmission into *k* parts of the same length. Each one of these sub-packets is then encoded with an error correction code and sent through the discrete channel [66]. Upon reception, if all the sub-packets from a sender are received with no collisions, the recovery of other packets coming from the same sender can be achieved by applying a maximum-a-posteriori (MAP) decoding scheme. In addition, the receiver also employs the interference cancellation scheme for reception from other senders. The work in [66] identifies the satellite network as a potential application for coded slotted Aloha (CSA).

The communications performance of the CSA protocol indicates a normalized throughput of S≃0.8 for a channel offered load of C≃0.84. The normalized load supported for a $PLR=10^{-3}$ is not reported in the literature.

Similar to CRDSA and other interference cancellation-based protocols, CSA is also energy efficient due to its low PLR for high channel loads. In terms of complexity, CSA is very similar to CRDSA, but it adds the difficulty of coding and decoding each packet with the model introduced in [66].

#### 5.3.4. Multi-Slots Coded Aloha (MuSCA)

This protocol generalizes the CRDSA protocol. By employing adequate error correction codes given a proper SNIR, multi slot coded Aloha (MuSCA) is able to decode packets even when there are collisions for all the transmissions in a frame [67]. In the example provided for CRDSA in Figure 11, MuSCA would have been able to successfully decode packets sent by users three and four, and received at slots two and five. The MuSCA protocol is designed for uplinks shared among a number of users in satellite systems [67].

MuSCA achieves a maximum normalized throughput of S=1.4 when the normalized channel load does not exceed 1.42. When the $PLR=10^{-3}$, MuSCA supports an offered load of C=1.22. Considering the very high efficiency of this protocol for normalized offered loads close to one, there are no need for retransmissions. In terms of complexity, MuSCA is very similar to CSA, since the difference between them is mainly in the coding mechanism.

#### 5.3.5. Enhanced Spread Spectrum Aloha (E-SSA)

**Figure 12:**
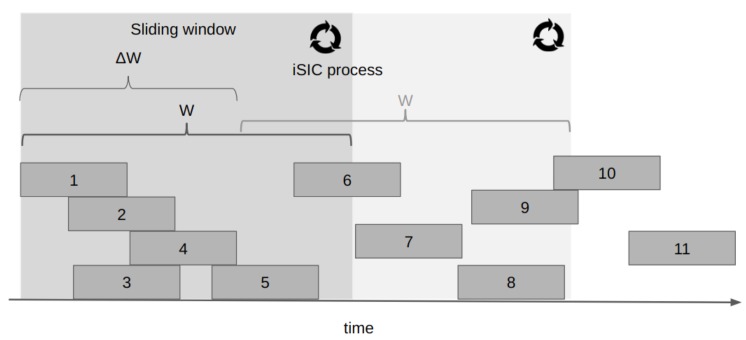
Example of operation of enhanced spread spectrum Aloha (E-SSA) protocol. The sliding window of length *W* is shifted in ΔW after performing the iterative process of interference cancellation (ISIC) in the current window. Figure adapted from [47].

This protocol is similar to SSA on the transmitter side. On the receiver side, enhanced spread spectrum Aloha (E-SSA) employs an iterative soft interference cancellation (ISIC) algorithm with a sliding window that captures the messages received on an unsynchronized channel. The main difference with the previous protocols is that E-SSA does not require sending multiple copies of packets, nor synchronization; thus, achieving a greater efficiency with a reduced complexity. The E-SSA protocol has been designed for integrated satellite/terrestrial mobile systems [5,68]. Figure 12 shows the operation of this protocol.

In terms of performance, the maximum normalized throughput reported for this protocol is S=1.2 for a channel load of C=1.25. When the $PLR=10^{-3}$, E-SSA is able to operate with a normalized load of C=1.12, assuming that the power imbalance of the transmitting nodes is equivalent. As opposed to SSA, in E-SSA, if a power imbalance of σ=3 dB is assumed among nodes, the performance improves considerably, achieving a normalized load of C=1.9 under a $PLR=10^{-3}$.

The interference-cancellation protocols presented in this section are designed mostly to solve the multiple access for the uplink of satellite systems. However, most of the control information required by the protocols is actually acquired/exchanged on the downlink. For example, a correct channel estimation is a key element for the proper operation of E-SSA. Such an estimation takes place over the downlink. Similarly, the code sharing process of CSA also takes place on the downlink. In half-duplex systems, the interference-cancellation based protocol’s feasibility requires careful examination.

Regarding the suitability of this category for IoT services, it can be mentioned that despite the great performance reported for these protocols, the complexity of their correct implementation makes them hard to adapt given the capabilities of CubeSats in a LEO orbit. Among the reviewed protocols, E-SSA stands out in performance compared to the others, and significantly reduces the complexity at the ground terminal; however, it is still highly demanding of resources on the nanosatellite side.

### 5.4. Hybrid Protocols

This category corresponds to protocols whose MAC mechanisms are based on a mix of protocols belonging to the other categories previously discussed in this section. The ones already mentioned and that also match this category are SS-CRDSA, MF-CRDSA, FC-TDMA, and R-Aloha; all of them employ a mix of fixed allocation techniques in conjunction with random access mechanisms.

#### Aloha-LoRa

Despite not being reported in satellite environments, another protocol that is worth mentioning due to its wide use in LPWA technologies is the one employed in LoRa. In this technology, the bandwidth is divided into several channels, where the number is dependent on the regulation (e.g., 13 channels for the industrial, scientific and medical (ISM) 902–928 MHz band under federal communications commission (FCC) regulation). In these channels, nodes transmit modulating the signal with the chirp spread spectrum (CSS) and make use of different spreading factors (SF). The channels are further divided into 6 subchannels (from SF = 7 to SF = 12). The MAC behavior depends on the LoRa class of device:

- Class A: The lowest power consumption type of device. Transmits data when is necessary using Pure Aloha. To receive messages from the gateway, a listening window is open after each transmission.
- Class B: For these devices there is a schedule to transmit, which is defined through beacons.
- Class C: These nodes are always in reception mode, except during transmission.

In the case of Class A nodes, the MAC protocol performs a fixed allocation of resources (in bandwidth and code) together with a random access scheme [14].

**Figure 13:**
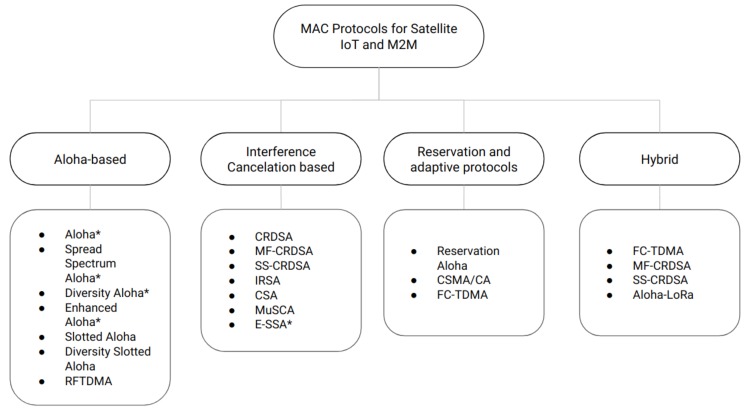
Taxonomy of random access protocols evaluated for satellite systems based on nanosatellites for IoT connectivity. Protocols with * indicate no time synchronization is needed.

To summarize our review, Figure 13 presents a taxonomy elaborated with the MAC protocols under study. Furthermore, the main characteristics and performance metrics when the MAC protocols are evaluated in a satellite environment are presented in Table 2.

## 6. Discussion and Open Research Challenges

In this section, we discuss the performance evaluation provided in Section 5 together with additional characteristics of the IoT scenario described in Section 3. Of the protocols reviewed, although several options offer a high throughput in terms of communications performance, such a good result is associated with a medium to high cost in terms of implementation complexity and energy consumption (see the first four protocols in Table 2). Our comparative analysis of protocols suggests that the Aloha-based are good candidates for the MAC layer in nanosatellites devoted to IoT connectivity. This is mainly due to their simplicity of implementation and their minimum hardware requirements. These protocols also report having a low sensitivity to delay.

In terms of network topology, the Aloha-based protocols can benefit from the power imbalance among nodes because of the so-called capture effect. In such a case, the receiver can correctly receive a packet with a high signal strength despite the existence of interference with other transmissions with lower power levels. Among the protocols in this category, the E-Aloha is the current solution for commercial telemetry satellite systems, has been extensively tested, and is operative. The performance of E-Aloha is similar to that of S-Aloha, with no need of synchronization. However, the main problem with E-Aloha is it may lack scalability for massive applications, due to its poor performance even for moderate traffic loads, as shown in Table 2. SS-Aloha, on the other hand, has much better performance than other Aloha-based protocols. However, this mechanism does not benefit from the capture effect; on the contrary, its performance falls to values similar to S-Aloha in a situation of power imbalance.

When examining the protocols based on interference cancellation (e.g., CRDSA and E-SSA in Table 2), the limitations in processing capacity in CubeSats, together with the adverse conditions to perform correct channel estimation in LEO orbits, make employing such protocols in the IoT study scenario infeasible. Other protocols in this category, such as MuSCA, CSA, and E-SSA, require the exchange of coordination or channel estimation information delivered in advance or through a separated channel, making it more demanding regarding channel resources.

As for protocols that require the carrier sensing mechanism, i.e., CSMA/CA, they have been shown to be highly inefficient given a topology with a moderate to a high number of hidden nodes, which will be a reasonably common scenario given the random distributions of ground sensors and devices serving a variety of IoT applications’ requirements. The performance of such protocols also decreases when the delays in transmissions among the nodes are highly uneven. In fact, the uneven delays are also critical for the operation of TDMA-inspired protocols such as R-Aloha and FC-TDMA. The reason is that each slot, to synchronize the channel, must incorporate a guard time of the order of the inequalities among the delays, which may result in a considerable waste of channel resources when the variability among delays is large. Moreover, despite the good performance reported for the throughput in FC-TDMA, it requires a specialized algorithm that varies dynamically the number of slots in each frame, which has not been determined in the definition of the protocol provided in [62]. In the case of R-Aloha, although it seems to have excellent performance, that only holds when reservations duration are such that the channel is always occupied; nevertheless, in the case of a large number of nodes, and short reservation times, the scalability of the protocol falls rapidly.

**Figure 14:**
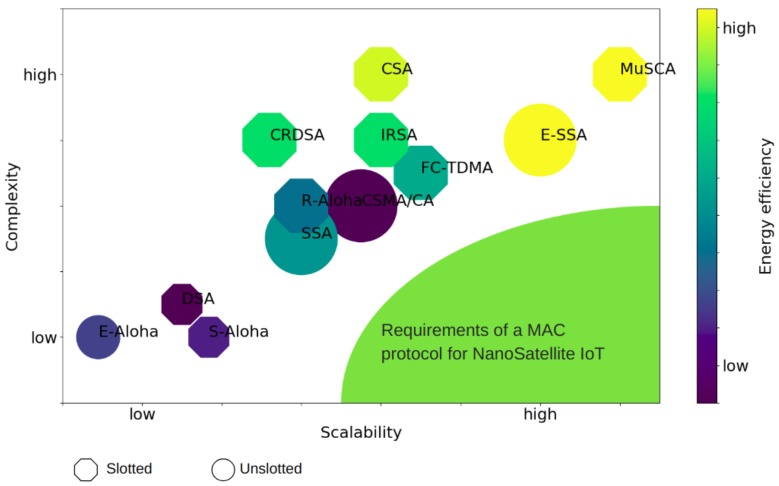
A visual comparison of the reviewed medium access control (MAC) protocols in the context of nanosatellite IoT scenarios.

As can be seen through comparative analysis, MAC protocol performance varies widely when examined in the context of CubeSats together with the characteristics of the IoT networks (number of nodes, nature of traffic, geographical distribution, etc.). A visual evaluation of the suitability of each protocol derived from our comparative analysis is shown in Figure 14. In the figure, the protocols are placed according to: (1) their fulfillment of IoT-related requirements such as the scalability (the x-axis), which relates to the communications performance when serving networks composed of a large number of nodes, the topology dependency (a larger-sized geometric figure enclosing each protocol indicates a larger dependency on terminal nodes locations and knowledge of network topology); and the energy efficiency (darker colors correspond to higher power consumption in the execution of the MAC protocol); and (2) their adaptation to the constraints of nanosatellite technology, in which case we evaluate their implementation complexity (the y-axis). A level of high complexity signifies the need of costly resources such as dynamic channeling, advanced channel estimation mechanisms, synchronization, etc.; and (once again) energy efficiency, since it is important to maintain the energy consumption on the spacecraft side within the nanosatellite capabilities.

To address the specific challenges derived from the utilization of nanosatellites for providing effective and cost-efficient IoT connectivity, we envision open research and implementation aspects from three perspectives: from the network protocols perspective, from the integration capabilities of CubeSats and from the evolution of the nanosatellites industry.

### 6.1. From the Network Protocols Perspective

When existing network protocols are evaluated in the context of nanosatellites technology for IoT connectivity, it is common to encounter difficulties in finding one protocol that meets all the requirements. In the case of MAC protocols, Figure 14 shows how an ideal performance zone, derived from the IoT scenario together with the CubeSat restrictions, is not yet met by any of the reviewed protocols, even though many had good performance in more traditional satellite scenarios. Additional research for MAC protocol design is needed to integrate aspects which can operate with low processing capacity demands and adapt to a variable and dynamic number of ground sensors and devices. Moreover, mitigation mechanisms should be considered for managing the high power imbalance conditions over the network links, the unequal delays derived from uneven link lengths, and the inability to provide high-quality channel estimations.

### 6.2. From the Integration Capabilities of CubeSat Connectivity with Other Wireless Technologies

The IoT ecosystem will benefit from a more integrated communications platform. In many cases, a global nanosatellite network integrated to an LPWA technology would boost the possibilities for improving connectivity at reduced costs. In recent years, there have been proposals for such a hierarchical architecture: the connected devices send data to an LPWA gateway, which in turn forwards data via the satellite network [9,69]. However, such an integration has not been explored with constellations of nanosatellites instead of traditional satellite networks. Additional research is needed to explore the requirements of compatibility in terms of MAC protocols, network architecture and united service patterns [10]. Another innovative line studied the behavior of an LPWA link to enable connectivity from the nanosatellite to a gateway on Earth [70,71]. Further research and experimentation will help understand and design an integrated platform that takes advantage of the different wireless technologies involved in these hybrid solutions.

### 6.3. From the Evolution of the Nanosatellites Industry

The enormous growth foreseen for the IoT market is highly related to the rapid evolution of low-cost wireless access technologies. In particular, with the introduction of LPWA technologies such as LoRa, Sigfox, and NB-IoT, to mention some, the massification of connected devices seems more plausible in the near future. Although nanosatellite connectivity is being identified as part of the IoT technologies ecosystem [6,7,8,9], it is still not considered as low-cost as to become part of the LPWA category [72,73]. The industry of nanosatellite construction and launching needs to keep evolving, and to evolve fast, to reduce costs even more and become another key player in the LPWA market.

## 7. Conclusions

The evolution of satellite systems and the introduction of CubeSats as low-cost satellite technology has made it possible to provide massive communications services for IoT applications, opening the opportunity for countries with no experience in space science and small corporations to participate competitively in the growing satellite communications market. However, the existent protocols in satellite technology, in particular for medium access control, were not designed with the IoT scenario, nor the low-cost technology constraints in mind.

This paper has presented a thorough review of MAC protocols designed for satellite environments, considering the specific characteristics of the IoT networks and applications together with the conditions of a wireless network served by CubeSats deployed in a low earth orbit. The study has shown that many of the reviewed protocols are not suitable for deployment in the scenario of interest, although they have been successfully implemented and deployed in other satellite systems. From the comparative evaluation, the protocols employing interference cancellation techniques are shown to have the best communications performance, but they behave poorly with regards to the demands of processing/channel resources and energy consumption. Furthermore, the Aloha-based protocols are good candidates for the MAC layer in nanosatellites devoted to IoT connectivity due to their simplicity of implementation and their minimum hardware requirements. However, these protocols report having poor communications performance when the traffic load—related to the growing number of expected nodes in the IoT—increases, and also when the delays vary greatly due to variable link lengths.

From our analysis, a better balance among performance, complexity, energy consumption, and sensitivity to topology should drive the design of future MAC protocols for nanosatellite IoT solutions. Aspects related to the network protocol design, the integration capabilities of CubeSat connectivity with other wireless technologies, and the evolution of the nanosatellites industry are some of the open challenges identified and discussed in this review.

## Figures and Tables

**Table 1:** A set of commercial constellations providing communication services as of 2018.

Company	Number of Satellites	Orbit	Services
Inmarsat	13	GEO	Broadband
Viasat	4	GEO	Broadband
Intelsat	52	GEO	Broadband
O3b	16	MEO	Broadband
Iridium	66	LEO	Voice, broadband
Globalstar	24	LEO	Voice, broadband

**Table 2:** Comparison of medium access control (MAC) protocols considering communications performance, complexity of implementation, energy efficiency, and the topology impact.

Protocols	$S_{\max}$	$C_{S_{\max}}$	$C_{\mathrm{PLR}=10^{-3}}$	Complexity	Energy Efficiency	Topology Impact *
MuSCA	1.4	1.42	1.22	high	high	different delays (−)
E-SSA	1.2	1.25	1.12	high	high	power imbalance (+)
FC-TDMA	1	1	-	medium	medium	variability in number of nodes (−),
						different delays (−)
R-Aloha	1	1	-	medium	medium	different delays (−)
CSA	0.8	0.84	-	high	high	different delays (−)
IRSA	0.8	0.85	0.7	high	high	different delays (−)
CSMA/CA	0.75	0.8	-	low	low	hidden nodes (−),
						different delays (−)
SS-Aloha	0.6	0.7	0.5	medium	medium	power imbalance (−)
CRDSA	0.52	0.65	0.05	high	medium	power imbalance (+),
						different delays (−)
S-Aloha	0.368	1	<$10^{-3}$	low	medium	power imbalance (+),
						different delays (−)
DSA	0.3	0.6	<$10^{-3}$	low	medium	power imbalance (+),
						different delays (−)
E-Aloha	0.09	0.1	<$10^{-3}$	low	medium	power imbalance (+)

${}^{*}$ (+) means the topology characteristics help improve the communications performance of the protocol, whereas (−) indicates a negative impact on performance.

## Abbreviations

The following abbreviations are used in this manuscript:

ACK	Acknowledgement
ADCS	Attitude determination and control system
ADS-B	Automatic dependent surveillance broadcast
AIS	Automatic identification system
CDMA	Code division multiple access
CRDSA	Contention resolution diversity slotted Aloha
CSA	Coded slotted Aloha
CSMA	Carrier sense multiple access
CSMA/CA	CSMA with collision avoidance
CSS	Chirp spread spectrum
CTS	Clear-to-send
DA	Diversity Aloha
DSA	Diversity slotted Aloha
E-SSA	Enhanced spread spectrum Aloha
ESA	European Space Agency
FCC	Federal communications commission
FC-TDMA	Fixed competitive TDMA
FDMA	Frequency division multiple access
GEO	Geosynchronous equatorial orbit
GLONASS	Global orbiting navigation satellite system
GNSS	Global navigation satellite system
GPS	Global positioning system
HEO	Highly elliptical orbit
ISIC	Iterative soft interference cancellation
IRSA	Irregular repetition slotted Aloha
IoT	Internet of things
ISM	Industrial, scientific and medical
LEO	Low Earth orbit
LPWA	Low power wide area
M2M	Machine to machine
MarCO	Mars Cube One
MAC	Medium access control
MEO	Medium Earth orbit
MF-CRDSA	Multi-frequency CRDSA
MuSCA	Multi slot coded Aloha
NASA	National Agency for Space Administration
OBC	On-board computer
OSI	Open system interconnection
PLR	Packet loss ratio
RFTDMA	Random frequency time division multiple access
RTS	Request-to-send
SNIR	Signal to noise plus interference ratio
SSA	Spread spectrum Aloha
SS-CRDSA	Spread spectrum CRDSA
SIC	Successive interference cancellation
TDMA	Time division multiple access
UCS	Union of Concerned Scientists
UNB	Ultra narrowband
